# Glycemic index and Glycemic load values

**DOI:** 10.12669/pjms.37.4.4555

**Published:** 2021

**Authors:** Jameel Ahmed, Musarrat Riaz, Rabia Imtiaz

**Affiliations:** 1 Jameel Ahmed, FRCP Professor and Chairman, Dean Faculty of Medicine, Baqai Medical University, Karachi, Pakistan; 2 Musarrat Riaz, FCPS Associate Professor, Department of Medicine, Baqai Medical University, Karachi, Pakistan; 3 Rabia Imtiaz, BS Clinical Dietician, Baqai Institute of Diabetology and Endocrinology, Baqai Medical University, Karachi, Pakistan

Dear Editor,

Postprandial hyperglycemia is a significant driver of common chronic diseases globally. In a single individual on a given day, carbohydrate-rich foods are the major determinant of Post Prandial Glycemia (PPG). Unfortunately, even after four decades of the publication of the first comprehensive list of Glycemic index (GI) values, many health professionals still believe that sugar and sugar-sweetened beverages have greater impact on PPG than starchy foods like bread and potatoes. This belief has no scientific basis. Most starchy foods have a GI greater than 70, while most sugary foods are less than 70,^1^ and on the whole, we eat twice as much energy in the form of starch as added sugar.^2^

Glycemic index has been widely used in the management of blood sugar levels among diabetes however; in Pakistan very little information regarding the GI of local foods is available. The GI is a ranking system that indicates how quickly a carbohydrate food raises blood glucose. This is determined by measuring the area under the curve in the two hours after the consumption of a test food. When describing the overall glycemic effects of a food and taking into consideration both the GI and the GL levels, it is often more practical to talk in generalities about a food’s “glycemic impact” instead of its specific GI or GL values.

There are a variety of factors that can account for the GI of different foods. Some of these characteristics are naturally occurring, whereas others are affected in commercialization or home preparation.^3^

## Physical form:

Generally, the more processed a food, the higher its GI.

## Food combinations:

When carbohydrate foods are eaten as part of a meal, the GI of the meal changes based on the average of all the GI values factored together.

## Cooking time:

Longer cooking times may increase the glycemic impact of a food by breaking down the starch or carbohydrate and allowing it to pass through the body more quickly when consumed.

## Acidity:

The more acidic a food is (e.g., pickled food or those containing vinegar or lemon juice), the lower the GI.

## Physical entrapment:

The fibrous coat around beans, seeds, and plant cell walls in whole grains acts as a physical barrier, slowing access of digestive enzymes to break down the carbohydrate. Thus, many whole grains and legumes have a lower GI.

## Protein/fat:

Adding protein or fat, which have minimal effects on glycemic excursions, to a high-GI food will decrease the GI of that food.

## Soluble fiber:

In general, the higher the food is in viscous or soluble fiber, the lower its GI will be.

Therefore, we did a clinical trial to assess the glycemic index and the glycemic load of 24 different Pakistani food items, which have not been studied. The food item tested were wheat Roti, Boiled rice, Potato, Fresh milk, Tetra pack milk, Domestic yogurt, Plain yogurt, Peas, Cucumber, Tomato, Cabbage, Onion, Cauliflower, Carrot, Dates, Papaya, Grapes, Boiled Eggs, Half fried Eggs, Salted Butter, Margarine, Cheese, Mars Chocolate and Glucose of same quantity that was 100 grams respectively. Twenty-four apparently healthy, fourth year medical students and house officers of Baqai Medical University (BMU) were recruited for this trial. Age range of the participants were between 22-25 years. There were 21 females and 3 male students with normal BMI and HbA1C and no truncal obesity. Participants were given a 100 grams carbohydrate portion of the test food items to ingest after a 10-12 hour fast overnight. In the morning, capillary blood samples were drawn from the fingers at 0 min, 15 minutes, 30 minutes, 60 and 120 minutes postprandially and the blood glucose level was determined.

**Fig:1:**
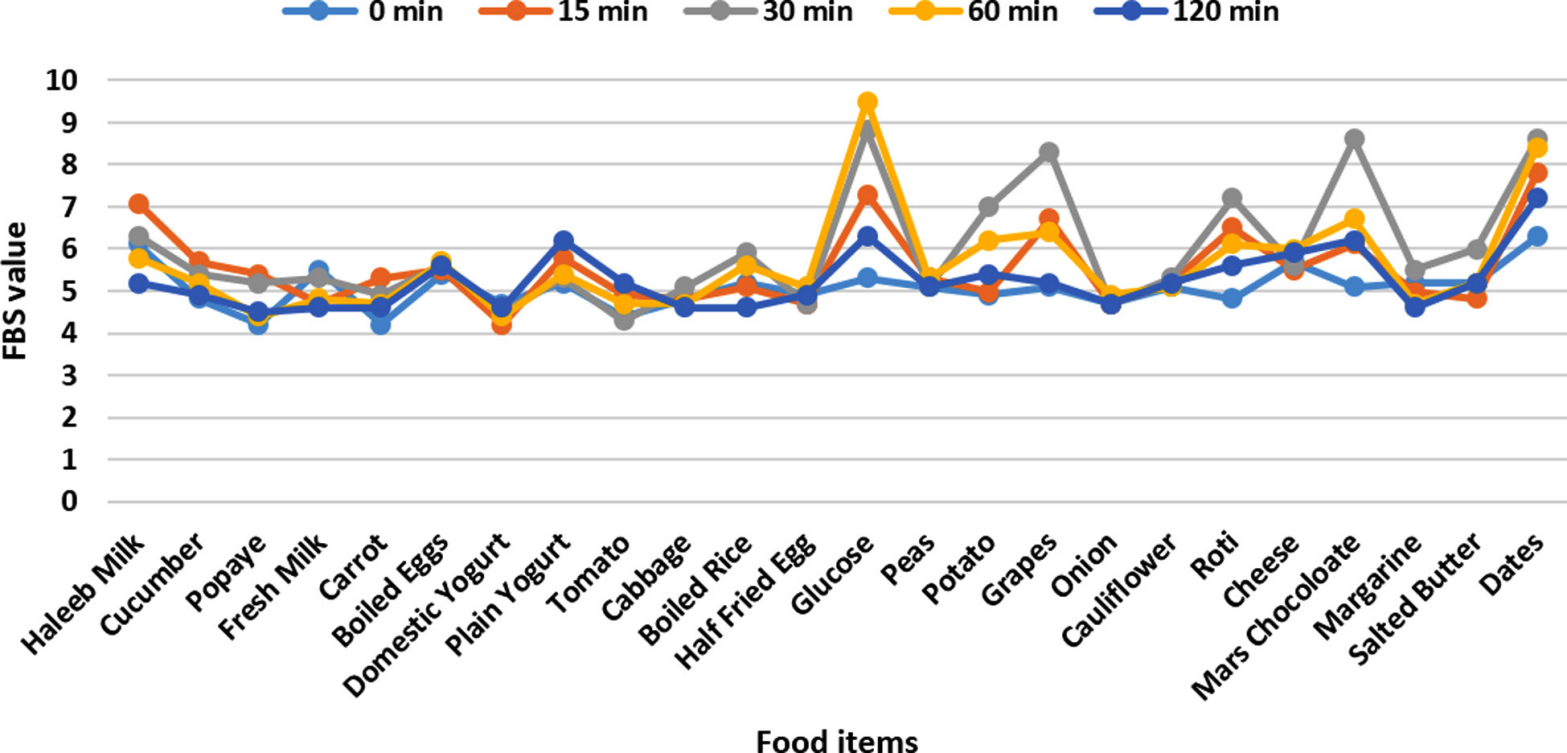
Food Graph.

The results showed that individuals respond to foods differently thus affecting the GI average values. The glycemic index has been categorized as low GI is 55, moderate GI is between 56-69 and high GI is > or = 70. Most of the food items understudy has moderate GI values ranging from 59 to 68. Although the Glycemic Index (GI) of glucose and mars chocolate were the highest i.e., GI 70. Moreover, talking specifically about the non-carbohydrate food items that were eggs and margarine, there were no changes seen in the PPG levels.

To conclude, the choice of carbohydrate-rich foods has the ability to influence post-prandial glycemia (glycemic index). Despite its endorsement by various health and governmental organizations, the GI concept remains undermined in Pakistan because of the lack of clinical trials and research studies. In light of the epidemic of conditions affecting glucose metabolism, it is strongly believed that the dietary GIycemic index and Glycemic Load should be communicated to the general public and health professionals through dietary guidelines, country-specific GI databases, food composition tables and food labels. Furthermore, local studies are required to confirm the public health implication of these findings.

The authors would like to acknowledge the support of Prof. Sikandar Ali Shaikh Ex, chairman, department of physiology, BMU for this study.
